# Social Wasps and Fruit Exploitation in Brazil: A Synthesis of Species Records, Resource Use, and Management Implications

**DOI:** 10.3390/insects17040409

**Published:** 2026-04-10

**Authors:** Bruno Corrêa Barbosa, Tatiane Tagliatti Maciel, Samanta Brito, Diego Rafael Gonzaga, Guy Smagghe, Rafael Dettogni Guariento

**Affiliations:** 1 Instituto de Biociências, Universidade Federal de Mato Grosso do Sul, Campo Grande 79070-900, Mato Grosso do Sul, Brazil; tatitagliatti@hotmail.com (T.T.M.); rafael.guariento@ufms.br (R.D.G.); 2 Instituto Nacional de Pesquisas da Amazônia, Manaus 69067-375, Amazonas, Brazil; brito.samanta25@gmail.com; 3 Instituto de Biodiversidade e Floresta, Universidade Federal do Oeste do Pará, Santarém 68040-255, Pará, Brazil; diego.gonzaga@gmail.com; 4 Department of Biology, Vrije Universiteit Brussel (VUB), 1050 Brussels, Belgium; guysma9@gmail.com; 5 Institute of Entomology, Guizhou University, Guiyang 550025, China

**Keywords:** citizen science, fruit exploitation, Hymenoptera, seasonality, wasp-plant interactions

## Abstract

Social wasps are frequently associated with fruit injuries in agroecosystems, but the diversity of species involved and the ecological context of these interactions remain poorly synthesized in Brazil. This study aimed to compile and analyze records of social wasp interactions with fruits in Brazil, combining opportunistic field observations, iNaturalist records, and published studies. In total, 19 field observations, 87 iNaturalist records, and 11 published studies were incorporated into the dataset, resulting in 2443 records involving 51 social wasp species in 10 genera and 36 plant species. Most records involved fruits still attached to the plant (1853; 75.8%), whereas 590 records (24.2%) were associated with fallen fruits. Large-bodied wasps were disproportionately associated with fruits on the plant, while small- and medium-sized species were more frequent on fallen fruits, and this association was significant (χ^2^ = 554.71; *p* < 0.001). Records also varied significantly throughout the year (χ^2^ = 5693.28; *p* < 0.001), with peaks in February, March, and July. The interactions were strongly concentrated in a few genera, especially *Polybia*, *Polistes*, and *Agelaia*, and in a few fruit species, particularly *Psidium guajava*, *Anacardium occidentale*, and *Mangifera indica*. Overall, the compiled evidence indicates that fruit exploitation by social wasps in Brazil is structured by body size, resource accessibility, and seasonality. However, because the study compiles heterogeneous records rather than directly measuring economic losses, the agricultural significance of these interactions remains context-dependent and requires further evaluation.

## Simple Summary

Social wasps are important insects in agroecosystems because they can help control herbivorous pests, but they may also interact with fruits and be perceived as harmful by growers. In Brazil, information on these interactions is still fragmented and based on isolated observations. In this study, we compiled records from field observations, iNaturalist, and published papers to provide a broader overview of social wasp interactions with fruits. We gathered 2443 records involving 51 social wasp species, 10 genera, and 36 plant species. Most records involved fruits still attached to the plant, and large-bodied wasps were more frequently associated with these fruits, whereas small- and medium-sized species were more common on fallen fruits. Records also varied throughout the year, with marked seasonal concentration. These results show that fruit exploitation by social wasps is influenced by body size, fruit accessibility, and seasonality. This information is valuable because it helps support more selective and ecologically based management practices and highlights the need for further studies quantifying the economic significance of these interactions.

## 1. Introduction

Hymenoptera play important functional roles in agroecosystems, acting as predators, pollinators, and regulators of insect populations. Among them, social wasps exhibit a highly generalist feeding habit throughout their life cycle, performing multiple ecological functions that directly influence agricultural productivity and pest dynamics [1]. Their trophic plasticity is associated with the functional division among castes and developmental stages: while larvae depend primarily on protein resources for growth, typically obtained through predation on other insects, adults rely mainly on energy-rich carbohydrate sources such as nectar, plant exudates, and ripe fruits [2]. This biological characteristic underlies both the ecosystem services and the potential risks associated with the presence of social wasps in agricultural environments.

The high protein demand of immature stages makes social wasps efficient predators of herbivorous insects, particularly Lepidoptera larvae, which represent some of the most economically important agricultural pests. These larvae are responsible for significant damage to leaves, flowers, and fruits in major crops in Brazil, including maize and sugarcane [3,4]. Consequently, social wasps have been increasingly recognized as potential biological control agents capable of regulating pest populations and contributing to reduced reliance on synthetic insecticides [5,6,7]. As native organisms well adapted to agricultural landscapes, their incorporation into integrated pest management (IPM) strategies represents a promising ecological approach aligned with sustainable agriculture and biodiversity conservation.

Beyond predation, some species of social wasps also participate in pollination processes, further expanding their functional relevance in agroecosystems [8,9]. Adult foraging behavior, which is essential for maintaining colony energy balance, frequently brings these insects into cultivated environments, particularly fruit production systems where carbohydrate-rich resources are abundant [1,10]. In these systems, social wasps may contribute simultaneously to pest suppression and, in some cases, to pollination services, reinforcing their role as multifunctional components of agricultural ecosystems.

However, the same foraging behavior that supports colony metabolism may also generate conflicts with agricultural production. Adults searching for carbohydrate sources may damage fruits directly on the plant, especially those with thin peels or at advanced stages of ripening. Such injuries can affect fruit quality and marketability, potentially resulting in economic losses for producers [11,12,13]. This dual role highlights an important ecological trade-off: organisms that contribute to pest suppression and ecosystem functioning may also act as agents of crop damage under certain production conditions.

Although these damages are typically localized and influenced by environmental factors, crop type, and management practices, they represent a relevant concern in fruit production systems, particularly for small-scale farmers whose income depends strongly on the commercial quality of harvested fruits [13]. Consequently, the presence of social wasps in agroecosystems raises important biological and applied questions: To what extent do these insects shift from beneficial predators to potential fruit pests, and under which ecological or management conditions does this transition occur? Addressing this issue is essential for the development of management strategies that balance the conservation of beneficial Hymenoptera with the mitigation of potential crop losses. From an applied perspective, understanding the contexts in which wasp–fruit interactions occur may support the design of targeted management approaches that preserve the biological control services provided by social wasps while minimizing negative impacts on fruit production. Such knowledge is particularly relevant for improving the integration of beneficial insects into IPM programs and for advancing ecologically based pest management strategies [5,6,13].

Despite the growing recognition of social wasps as beneficial organisms in agricultural systems, information on their interactions with fruits in Brazil remains fragmented in the scientific literature. Existing evidence is largely derived from isolated observations and localized case studies, limiting broader assessments of the diversity of species involved, the types of fruits exploited, and the ecological context in which these interactions occur [11,12,13]. In this context, the present study aimed to compile and analyze records of social wasp interactions with fruits in Brazil by integrating opportunistic field observations, iNaturalist records, and published studies. Specifically, we evaluated the diversity of social wasp and plant species involved, the position of fruits at the time of interaction, and the seasonal distribution of records. We also tested whether fruit position was associated with wasp body size, expecting larger species to be more frequently associated with fruits still attached to the plant, whereas small and medium-sized species would occur predominantly on fallen fruits.

## 2. Materials and Methods

The data used in the present study were obtained through three complementary sources: direct field observations, records from the iNaturalist platform, and a comprehensive review of the scientific literature. In total, 19 direct field observations, 87 records from iNaturalist, and 11 published studies were incorporated into the dataset. For studies obtained from the literature, individual occurrences of wasp–fruit interactions reported by the authors were extracted and included as independent records in the dataset. The integration of these data sources enabled the inclusion of information from different ecological contexts, spatial scales, and sampling conditions, providing a broader overview of wasp–fruit interactions in Brazil.

### 2.1. Field Observations

Opportunistic field records of interactions between social wasps and fruits were obtained during 2024 and 2025 in three Brazilian municipalities, Campo Grande, Mato Grosso do Sul state (7 records), Manaus, Amazonas state (4 records), and Altos, Piauí state (8 records), totaling 19 field observations. Interactions were recorded ad libitum whenever wasps were observed visiting, feeding on, or removing resources from fruits. For each record, we noted the social wasp species involved, the associated plant species, and the position of the fruit at the time of interaction (attached to the plant or fallen on the ground).

Wasps were collected with an entomological net and stored in microtubes containing 70% ethanol. Specimens were identified to genus level using Somavilla and Carpenter [14] and, whenever possible, to the lowest taxonomic level based on Richards [15]. Plant identifications and nomenclature were checked using Flora e Funga do Brasil [16] and Plants of the World Online [17]. The collected material was deposited in the Zoological Collection of the Universidade Federal de Mato Grosso do Sul (UFMS) and in the Invertebrate Collection of the Instituto Nacional de Pesquisas da Amazônia (INPA).

**Figure 1:**
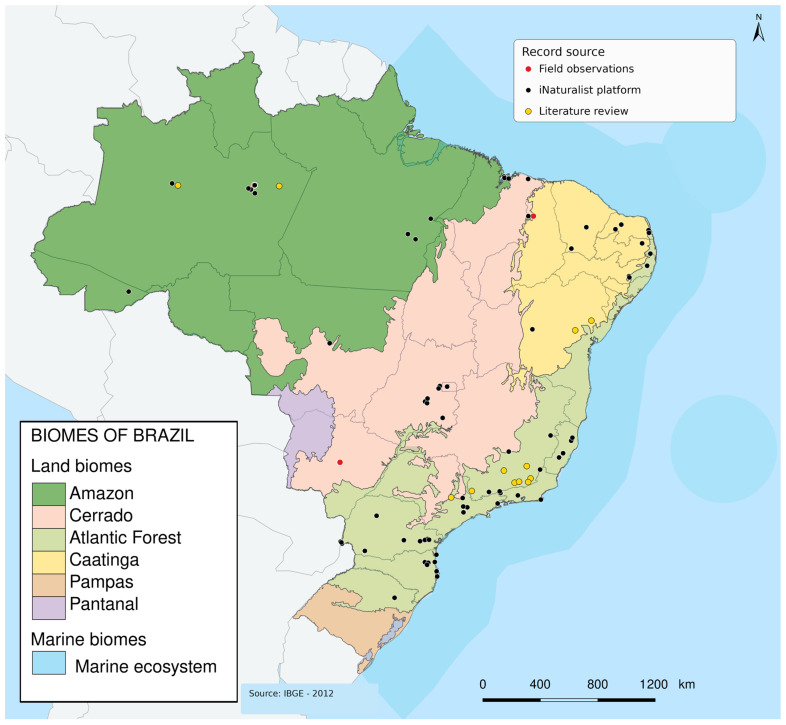
Geographic distribution of records of social wasp interactions with fruits in Brazil, based on field observations, iNaturalist records, and literature review.

Field records were obtained at the following localities: Campo Grande, Mato Grosso do Sul, Brazil (20°30′40.80″ S, 54°37′15.60″ W; 20°30′08.26″ S, 54°36′42.58″ W); Manaus, Amazonas, Brazil (3°06′06.85″ S, 59°58′34.48″ W; 3°05′43.64″ S, 59°59′22.77″ W); and Altos, Piauí, Brazil (5°2′24″ S, 42°27′41″ W). These localities are shown in Figure 1.

### 2.2. iNaturalist Platform

Records of interactions between social wasps and fruits were obtained from the citizen science platform iNaturalist (https://www.inaturalist.org) through manual screening of public observations. Observations assigned to Polistinae and filtered to Brazil were inspected individually during data compilation for this study, considering records available on the platform up to December 2025. Because the platform does not allow a standardized keyword-based search equivalent to bibliographic databases, records were manually located by browsing Brazilian observations and selecting those that clearly documented interactions between social wasps and fruits.

Only records with photographic evidence sufficient to confirm both the interaction and the identity of the organisms involved were included. Specifically, accepted records allowed identification of both the social wasp and the plant at least to genus level. Records were excluded when either the wasp or the plant could not be reliably identified, or when the interaction with fruit was unclear. Taxonomic identifications were checked by coauthors with expertise in social wasps and plants, using the same references adopted for the field records. Social wasps were identified following Somavilla and Carpenter [14] and Richards [15], whereas plant identifications and nomenclature were checked using Flora e Funga do Brasil [16] and Plants of the World Online [17]. The URLs of all iNaturalist records included in the dataset are provided in Supplementary Table S1.

### 2.3. Literature Review

A literature survey was conducted to compile published records of interactions between social wasps and fruits in Brazil. Searches were performed in the Scientific Electronic Library Online (SciELO), Scopus, Web of Science, and Google Scholar databases, considering publications available up to December 2025. Search terms included “vespa*”, “wasp*”, “marimbondo*”, “cabas*”, or “Vespidae”, combined with “fruto*”, “fruta*”, or “fruit*”, along with terms related to damage or predation. The asterisk (*) was used as a wildcard to capture different word endings and variations in the root terms.

The retrieved studies were initially screened based on title and abstract, and potentially relevant papers were then assessed in full text. Additional studies were also identified from the reference lists of the selected papers.

Only studies that explicitly reported interactions between social wasps and fruits consumed in Brazil were included. Studies addressing other food resources, those that did not specify Brazil, those aggregating data from multiple countries without allowing identification of Brazilian records, and those that did not identify at least the wasp genus or the type of fruit were excluded. For each eligible study, the interactions reported by the authors were extracted and incorporated into the dataset. When studies presented abundance data or multiple observations of the same wasp–fruit association, these were treated as individual records rather than collapsing each publication into a single record. This procedure allowed the dataset to reflect the frequency of interactions reported in the original sources.

### 2.4. Classification of Social Wasp Body Size

Body size of social wasps was treated as a categorical functional trait to interpret patterns of fruit foraging. Species were classified into small, medium, and large size categories based on direct measurements of body length obtained from specimens deposited in the Invertebrate Collection of the National Institute of Amazonian Research and the Zoological Collection of the Federal University of Mato Grosso do Sul, complemented by information from the literature.

Wasps were classified into three body-size categories based primarily on mean body length. Species with a mean body length < 15 mm were categorized as small. Species with a mean body length between 15 and 20 mm were classified as medium-sized, including representatives of *Agelaia* and *Mischocyttarus*. Although some species within these genera may exceed 20 mm in length, their comparatively slender morphology and lower body robustness justified their inclusion in the medium-sized category, as they lack the robust body form characteristic of large-bodied genera.

Species with a mean body length > 20 mm were classified as large. This category included representatives of *Synoeca*, *Polistes*, and *Apoica*, as well as large-bodied species of *Polybia*, namely *Polybia chrysothorax* (Lichtenstein, 1796), *Polybia dimidiata* (Olivier, 1791), *Polybia ignobilis* (Haliday, 1836), *Polybia jurinei* de Saussure, 1854, *Polybia liliacea* (Fabricius, 1804), *Polybia striata* (Fabricius, 1787), and *Polybia sericea* (Olivier, 1792).

This classification was not intended to employ continuous measurements or detailed morphometric analyses, but rather to assess whether functional differences associated with body size were related to the position of the exploited fruit, either on the plant or fallen on the ground.

### 2.5. Statistical Analysis

To test the association between social wasp characteristics, particularly body size, and fruit position (on the plant or fallen), a chi-square test of independence was performed. In addition, descriptive analyses were conducted to evaluate genus-level dominance based on total record percentages, as well as species seasonality throughout the sampling period. All statistical analyses were performed using R software version 4.4.0 [18], adopting a significance level of *p* < 0.05.

### 2.6. Bipartite Interaction Network

To visualize interaction patterns between social wasps and fruit species, a quantitative bipartite interaction network was constructed. The matrix included all wasp species positioned on the left and all fruit species on the right. Interaction frequency was used as a weighting factor, and link thickness was proportional to the number of recorded interactions for each species pair. The network was generated in R using the bipartite package [19] and was used for descriptive visualization of interaction patterns.

## 3. Results

A total of 2443 records of social wasp interactions with fruits were compiled in this study, including 19 field observations, 87 iNaturalist records, and 2337 records extracted from 11 published studies. Of these, 1853 records (75.8%) involved fruits still attached to the plant, whereas 590 records (24.2%) involved fallen fruits. The dataset included 51 social wasp species distributed across 10 genera and 36 plant species (Table 1).

The distribution of records revealed a strong concentration within a few genera. *Polybia* (*n* = 1077; 44.1%), *Polistes* (*n* = 546; 22.3%), and *Agelaia* (*n* = 471; 19.3%) together accounted for 85.7% (*n* = 2094) of all compiled records. Within these three dominant genera, 74% (*n* = 1549) of the records involved fruits still attached to the plant, whereas 26% (*n* = 545) were associated with fallen fruits.

Among the dominant genera, *Polybia* presented the highest absolute number of records, with a predominance of injuries in fruits on the plant (78.4%; *n* = 844) compared with fallen fruits (21.6%; *n* = 233). This pattern was strongly influenced by *P. sericea* (*n* = 326; 38.6%) and *P. ignobilis* (*n* = 198; 23.5%), both large-bodied species frequently observed perforating the fruit epidermis to access internal resources. A similar pattern was observed for *Polistes* and *Synoeca*, which showed a strong association with the active exploitation of fruits still attached to the plant, representing 96.3% (*n* = 526) and 91.9% (*n* = 181) of their records, respectively. In both genera, individuals were also observed directly perforating the fruit epidermis.

In contrast, *Agelaia* exhibited a distinct pattern of resource use. Only 38% (*n* = 179) of its records involved fruits on the plant, whereas 62% (*n* = 292) were associated with fallen fruits. No individuals of this genus were recorded perforating the fruit epidermis.

These behavioral differences were reflected in the analysis of the relationship between body size and fruit position. A significant association was found between wasp body size and fruit position (χ^2^ = 554.71; *p* < 0.001). Large-bodied species, including all evaluated species of *Apoica*, *Polistes*, and *Synoeca*, as well as *P. chrysothorax*, *P. dimidiata*, *P. ignobilis*, *P. jurinei*, *P. liliacea*, *P. striata*, and *P. sericea*, accounted for 1269 records of injuries in fruits still attached to the plant, corresponding to 68.1% of all records in this category (*n* = 1853). In contrast, these same species represented only 139 records in fallen fruits, equivalent to 23.2% of the total records for this resource type (*n* = 590).

Conversely, medium- and small-bodied wasps, including all species of *Agelaia*, *Mischocyttarus*, *Parachartergus*, *Protonectarina*, and *Protopolybia*, were predominantly associated with records involving fallen fruits. These species accounted for 584 records in fruits still attached to the plant (31.9% of that category) but concentrated most records in fallen fruits, representing 76.8% of occurrences (*n* = 459). Only two records involved smaller species, *Polybia occidentalis* (Olivier, 1791) and *Polybia scutellaris* (White, 1841), associated with fruit perforation.

In addition to taxonomic and functional patterns, the temporal analysis of the 2443 records indicated significant variation in the occurrence of fruit injuries throughout the year, rejecting the hypothesis of a uniform distribution (χ^2^ = 5693.28; *p* < 0.001). The observed pattern was bimodal, with two periods of higher concentration of records. The highest number of occurrences was recorded in July, accounting for 37.4% of the records (*n* = 914), followed by March (29.2%; *n* = 713) and February (21.3%; *n* = 521). Considering seasonal distribution, the autumn and winter period in Brazil, from March to August, accounted for 75.8% of all records.

**Figure 2:**
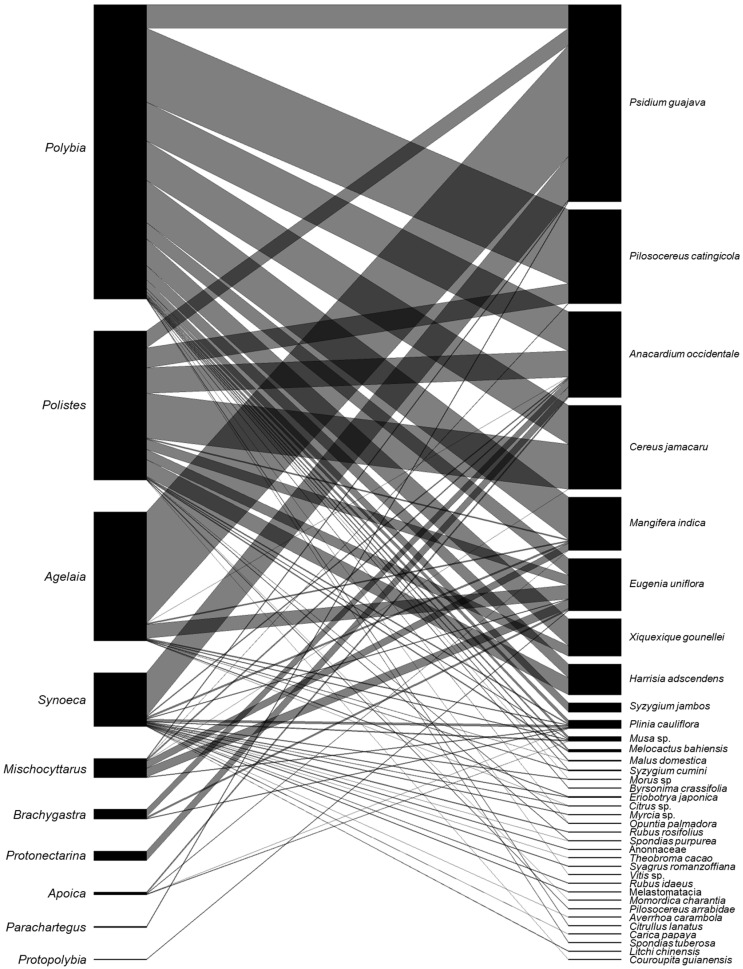
Bipartite network showing interactions between social wasps and fruiting plants. Link thickness represents the frequency of interactions. On the left are 51 species of social wasps distributed across 10 genera, and on the right are 36 fruit species exploited by social wasps.

There was also a strong asymmetry in the abundance of interactions between social wasps and plants, with a few plant species concentrating most records (Figure 2). *Psidium guajava* L. (Myrtaceae), *Anacardium occidentale* L., and *Mangifera indica* L. (Anacardiaceae) showed the highest number of records and the greatest number of connections (*n* = 1228), indicating frequent foraging by multiple social wasp species (Figure 2). In contrast, most other plant species exhibited few connections and low record frequencies. This pattern indicates that, despite the high diversity of exploited plants, wasp–fruit interactions were strongly concentrated within a restricted subset of plant species.

## 4. Discussion

The compiled records indicate that fruit exploitation by social wasps in Brazil is structured by taxonomic concentration, body size, fruit accessibility, and seasonality. Interactions were concentrated in a limited subset of wasp genera and plant species, and the position of the fruit at the time of interaction was strongly associated with wasp body size. By integrating field observations, iNaturalist records, and published studies, this synthesis provides a broader overview of the ecological patterns underlying wasp–fruit interactions in Brazil.

One of the most evident patterns was the strong asymmetry in the plant-wasp interaction network. The concentration of records in *P. guajava*, *A. occidentale*, and *M. indica* suggests that fruit exploitation by social wasps is largely driven by resource availability and predictability rather than strict feeding preference. These fruit species are widely cultivated and commonly found in anthropogenic landscapes, where they provide abundant carbohydrate resources over extended fruiting periods [28,29]. Because social wasps are opportunistic generalists capable of exploiting diverse food sources, they are more likely to encounter and repeatedly exploit abundant and predictable resources in agricultural environments [29,30,31]. Consequently, the asymmetry observed in the interaction network likely reflects a resource availability bias, in which interaction records concentrate on plant species that are more frequently available in productive systems, rather than indicating exclusive preference for particular fruit species.

Another central finding of this study is the strong association between functional traits and patterns of fruit exploitation, particularly regarding body size. Larger species, such as those belonging to *Polybia*, *Polistes*, and *Synoeca*, were predominantly associated with fruits still attached to the plant and were frequently observed perforating the fruit epidermis. This ability allows these species to access internal carbohydrate resources even when fruits are intact, effectively expanding the spectrum of exploitable food sources. Such behavior reduces dependence on previously damaged fruits and may contribute to niche partitioning among co-occurring species, decreasing direct interspecific competition for carbohydrate resources [32,33]. From an ecological perspective, this finding supports the hypothesis that body size functions as a key determinant of resource access in social wasps, influencing the types of substrates that can be exploited during foraging.

In contrast, we found that smaller and medium-sized species showed a markedly different pattern, being predominantly associated with fallen fruits. This pattern suggests a more opportunistic strategy in which wasps exploit resources that have already been exposed through natural processes, physical damage, or the activity of other organisms. Such opportunistic exploitation is consistent with the generalist feeding habits described for many species of the subfamily Polistinae [1,5]. The distinction between active fruit perforators and opportunistic exploiters therefore reflects a form of functional differentiation within the social wasp community, structured by morphological and behavioral constraints.

However, resource accessibility is not determined exclusively by wasp traits. Fruit morphology also plays an important role in shaping these interactions. Structural features such as peel thickness, firmness, and mechanisms of natural dehiscence may restrict access to carbohydrate resources while fruits remain attached to the plant [34,35]. In some cases, fruits may become more accessible to insects after abscission, when loss of firmness and disruption of skin integrity increase tissue exposure [36,37]. Although such cases were relatively uncommon in our dataset, they illustrate that patterns of fruit exploitation arise from the interaction between consumer traits and resource characteristics. In other words, the realized niche of social wasps in fruit exploitation emerges from the combined effects of functional traits of the consumer and structural properties of the resource.

Although social wasps are generally described as opportunistic generalist predators [1,5], the comparative analysis conducted here revealed consistent differences among genera in their use of fruit resources. The genus *Agelaia* provides a clear example of an opportunistic strategy. Individuals were predominantly recorded on fallen fruits, consistent with the well-known scavenging behavior of this genus [38], which has also been documented in association with carrion exploitation [39]. This pattern suggests that *Agelaia* species frequently exploit resources already available in the environment rather than actively modifying them to gain access.

In contrast, the genus *Synoeca* exhibited a strong and consistent association with fruit exploitation. All recorded species of this genus interacted with fruits, and the genus presented the highest mean number of fruit-use records per species. The recurrence of this pattern across congeneric species indicates a relatively stable ecological association with carbohydrate-rich plant resources. This observation does not imply strict dietary specialization but rather reflects functional specialization, defined as the repeated and consistent use of a particular resource within a broader generalist diet [40,41]. Such functional tendencies are relevant for understanding how different social wasp taxa integrate into agroecosystem trophic networks.

Seasonality emerged as another key factor structuring fruit exploitation by social wasps. The bimodal pattern observed, with higher frequencies during the autumn and winter months in Brazil, is consistent with previously documented seasonal dynamics in social wasp foraging behavior [1,42]. This period coincides with the fruiting of several plant species and with reductions in prey availability due to leaf loss in many plants [43]. Under these conditions, social wasps appear to intensify the use of fruits as alternative carbohydrate sources, demonstrating the high dietary plasticity characteristic of generalist foragers. This seasonal shift in resource use has important ecological implications. It suggests that fruit exploitation by social wasps is partially driven by temporal fluctuations in the availability of other resources, particularly insect prey. Consequently, fruit foraging may represent an adaptive response that allows colonies to maintain energy intake during periods when protein sources are less abundant. From a sociobiological perspective, this flexibility is consistent with the colony-level nutritional requirements of social wasps, in which adult workers must balance the acquisition of carbohydrates for colony maintenance with protein resources necessary for larval development.

Taken together, these findings suggest that wasp–fruit interactions in Brazil are shaped by identifiable ecological factors rather than being uniformly distributed across taxa, resources, or seasons. In this sense, the dataset helps clarify the conditions under which social wasps are more frequently recorded exploiting fruits, while also indicating that these interactions should be interpreted in light of resource accessibility and temporal variation.

### 4.1. Management Implications

The patterns documented in this study suggest that management of wasp–fruit interactions in orchards should be selective and context-specific. Because large-bodied species were more frequently associated with fruits still attached to the plant, preventive measures directed at intact fruits, such as bagging, physical barriers, or localized deterrents, may help reduce direct exploitation without requiring colony elimination. These strategies are more consistent with ecologically based pest management than indiscriminate suppression, particularly because social wasps also provide relevant ecosystem services in agricultural systems [44,45].

For genera predominantly associated with fallen fruits, especially *Agelaia*, sanitation practices are likely to be more appropriate. The removal of fallen fruits, fermenting organic matter, and previously damaged fruits may reduce the availability of accessible carbohydrate resources and, consequently, the frequency of wasp visits in orchards [46,47]. This distinction is important because not all records represent the same type of interaction: some involve direct perforation of intact fruits, whereas others reflect opportunistic exploitation of already accessible resources.

Seasonal variation should also be considered when planning management actions. Because fruit exploitation was concentrated in specific months, preventive actions targeting fruits on the plant and sanitation practices targeting fallen fruits should be prioritized during periods of higher interaction frequency. More broadly, the present synthesis indicates that management decisions should be based on the type of interaction observed, the taxa involved, and the production context, rather than on a generalized assumption that all social wasps act as fruit pests.

Environmental education may also help reduce unnecessary colony destruction. Farmers often interpret any wasp presence on fruits as evidence of direct damage, although some records may involve opportunistic feeding or the use of fruits as foraging sites associated with prey capture. Improving recognition of these different interaction contexts may support more selective and ecologically informed decisions in fruit production systems.

### 4.2. Limitations and Future Directions

Despite providing the most comprehensive synthesis to date of fruit injuries caused by social wasps in Brazil, this study also presents some limitations. First, the dataset integrates records obtained through different sampling approaches, including opportunistic observations, citizen science data, and literature reports. Although this integration expands spatial and temporal coverage, it may introduce sampling biases related to observation effort and reporting frequency. Second, the study primarily documents the occurrence of interactions, rather than directly quantifying the magnitude of economic losses associated with fruit damage. As a result, the actual economic significance of these injuries remains uncertain and may vary substantially among crops, regions, and production systems.

Therefore, we suggest that future research should focus on controlled field studies capable of quantifying the real impact of fruit damage caused by social wasps under different agricultural conditions. In particular, evaluating economic thresholds for intervention would represent a critical step toward integrating these insects into evidence-based pest management frameworks. Another promising direction involves examining the cost–benefit balance between fruit damage and the ecosystem services provided by social wasps, particularly their role in controlling herbivorous pests. Such assessments would allow a more holistic evaluation of their ecological value in agricultural systems. Finally, additional studies exploring the behavioral and physiological mechanisms underlying fruit exploitation may provide deeper insights into how environmental conditions, colony nutritional demands, and resource availability interact to shape foraging decisions in social wasps.

## 5. Conclusions

This study provides a broad synthesis of social wasp interactions with fruits in Brazil by integrating field observations, iNaturalist records, and published studies. The compiled evidence shows that fruit exploitation is not randomly distributed, but is structured by body size, resource accessibility, and seasonality. Large-bodied wasps were more frequently associated with fruits still attached to the plant, whereas small- and medium-sized species were more often recorded on fallen fruits, indicating functional differences in how these resources are accessed. The results also show that these interactions are concentrated in a limited subset of wasp genera and fruit species, reinforcing the importance of ecological context in shaping foraging patterns.

Although the dataset does not allow direct estimation of economic losses, it supports the view that social wasp interactions with fruits should not be interpreted uniformly as pest activity. Instead, management decisions should consider the type of interaction, the taxa involved, and the ecological services provided by these insects in agroecosystems.

## Figures and Tables

**Table 1:** Recorded interactions between social wasps and fruiting plants, including the total number of records and their respective data sources.

Wasp Species	Plant Species	Data Source
*Agelaia angulata* (Fabricius, 1804)	*Citrus* sp. (1); *Musa* sp. (2)	iNaturalist [Table S1, record 1–3]
*Agelaia multipicta* (Haliday, 1836)	*Eugenia uniflora* (48); *Malus domestica* (1); *Plinia cauliflora* (1)	De Souza et al. [20]; Souza et al. [21]; iNaturalist [Table S1, record 4]
*Agelaia pallipes* (Olivier, 1792)	*Anacardium occidentale* (1); *Eriobotrya japonica* (1); *Musa* sp. (1); *Psidium guajava* (406); *Syzygium cumini* (1)	Renne et al. [22]; iNaturalist [Table S1, record 5–7]; Field observations (this study)
*Agelaia* sp.	*Mangifera indica* (1)	Field observations (this study)
*Agelaia testacea* (Fabricius, 1804)	*Plinia cauliflora* (1)	Oliveira et al. [23]
*Agelaia vicina* (de Saussure, 1854)	*Plinia cauliflora* (1); *Eugenia uniflora* (2); *Mangifera indica* (3)	De Souza et al. [20]; Souza et al. [21]; Barbosa et al. [12]
*Apoica flavissima* Van der Vecht, 1972	*Mangifera indica* (1)	iNaturalist [Table S1, record 8]
*Apoica pallens* (Fabricius, 1804)	*Anacardium occidentale* (5); *Plinia cauliflora* (1)	Oliveira et al. [23]; Field observations (this study)
*Apoica* sp.	*Musa* sp. (1)	iNaturalist [Table S1, record 9]
*Brachygastra lecheguana* (Latreille, 1804)	*Anacardium occidentale* (27); *Eugenia uniflora* (7); *Plinia cauliflora* (2)	De Souza et al. [20]; Santos & Presley [24]; Souza et al. [21]; Oliveira et al. [23]
*Mischocyttarus araujo* Zikán, 1949	*Mangifera indica* (3)	Barbosa et al. [12]
*Mischocyttarus cassununga* (R. von Ihering, 1903)	*Eugenia uniflora* (29); *Mangifera indica* (22)	Souza et al. [21]; Barbosa et al. [12]
*Mischocyttarus consobrinus* de Saussure, 1858	*Plinia cauliflora* (1)	Oliveira et al. [23]
*Mischocyttarus drewseni* de Saussure, 1857	*Eugenia uniflora* (1); *Psidium guajava* (4)	Souza et al. [21]; Renne et al. [22]
*Mischocyttarus lanei* Zikán, 1949	*Cereus jamacaru* (1); *Pilosocereus catingicola* (1)	Santos et al. [25]
*Mischocyttarus socialis* (de Saussure, 1854)	*Eugenia uniflora* (2)	Souza et al. [21]
*Mischocyttarus* sp.	*Anacardium occidentale* (4); *Plinia cauliflora* (1)	Santos & Presley [24]; Oliveira et al. [23]
*Parachartergus pseudapicalis* Willink, 1959	*Psidium guajava* (3)	Renne et al. [22]
*Polistes actaeon* Haliday, 1836	*Eugenia uniflora* (4)	Souza et al. [21]
*Polistes billardieri* Fabricius, 1804	*Cereus jamacaru* (1); *Harrisia adscendens* (2); *Pilosocereus catingicola* (4); *Xiquexique gounellei* (3)	Santos et al. [25]
*Polistes canadensis* (Linnaeus, 1758)	*Anacardium occidentale* (52); *Cereus jamacaru* (142); *Eugenia uniflora* (1); *Harrisia adscendens* (8); *Melocactus bahiensis* (1); *Opuntia palmadora* (1); *Pilosocereus catingicola* (32); *Psidium guajava* (1), *Spondias purpurea* (1); *Xiquexique gounellei* (29)	Santos et al. [25]; Santos & Presley [24]; iNaturalist [Table S1, record 10–13]
*Polistes carnifex* (Fabricius, 1775)	*Psidium guajava* (1)	iNaturalist [Table S1, record 14]
*Polistes ferreri* (de Saussure, 1853)	*Plinia cauliflora* (1); *Psidium guajava* (28)	Renne et al. [22], Oliveira et al. [23]
*Polistes lanio* (Fabricius, 1775)	*Pilosocereus arrabidae* (1)	iNaturalist [Table S1, record 15]
*Polistes simillimus* Zikán, 1948	*Cereus jamacaru* (6); *Eugenia uniflora* (11); *Melocactus bahiensis* (2); *Pilosocereus catingicola* (9); *Plinia cauliflora* (2); *Psidium guajava* (1); *Xiquexique gounellei* (1)	Santos et al. [25]; De Souza et al. [20]; Souza et al. [21]; Renne et al. [22]; Oliveira et al. [23]
*Polistes* sp.	*Musa* sp (1)	iNaturalist [Table S1, record 16]
*Polistes versicolor* (Olivier, 1792)	*Anacardium occidentale* (43); *Cereus jamacaru* (14); *Eugenia uniflora* (21); *Harrisia adscendens* (52); *Mangifera indica* (4); *Melocactus bahiensis* (1); *Momordica charantia* (1); *Pilosocereus catingicola* (27); *Plinia cauliflora* (1); *Psidium guajava* (30) *Xiquexique gounellei* (5)	Santos et al. [25]; Santos & Presley [24]; Souza et al. [21]; Barbosa et al. [12]; Renne et al. [22]; Oliveira et al. [23]; iNaturalist [Table S1, record 17]
*Polybia bifasciata* de Saussure, 1854	*Eugenia uniflora* (1); *Mangifera indica* (8)	Souza et al. [21]; Barbosa et al. [12]
*Polybia chrysothorax* (Lichtenstein, 1796)	*Mangifera indica* (1)	iNaturalist [Table S1, record 18]
*Polybia dimidiata* (Olivier, 1792)	*Byrsonima crassifolia* (1); *Psidium guajava* (1)	Lourido et al. [13]
*Polybia fastidiosuscula* de Saussure, 1854	*Eugenia uniflora* (1); *Mangifera indica* (15)	Souza et al. [21]; Barbosa et al. [12]
*Polybia ignobilis* (Haliday, 1836)	*Anacardium occidentale* (55); *Cereus jamacaru* (33); *Eugenia uniflora* (1); *Harrisia adscendens* (18); *Mangifera indica* (34); *Melocactus bahiensis* (2); *Musa* sp. (2); *Pilosocereus catingicola* (35); *Plinia cauliflora* (5); *Psidium guajava* (19); *Spondias tuberosa* (1); *Xiquexique gounellei* (46)	Santos et al. [25]; Santos & Presley [24]; De Souza et al. [20]; Souza et al. [21]; Barbosa et al. [12]; Oliveira et al. [23]; Renne et al. [22]; iNaturalist [Table S1, record 19–24]; Field observations (this study)
*Polybia jurinei* de Saussure, 1854	*Citrullus lanatus* (1); *Eugenia uniflora* (1); *Mangifera indica* (19); *Musa* sp. (1); *Plinia cauliflora* (1); *Psidium guajava* (38); *Theobroma cacao* (1)	De Souza et al. [20]; Souza et al. [21]; Barbosa et al. [12]; Renne et al. [22]; iNaturalist [Table S1, record 25–28]
*Polybia liliacea* (Fabricius, 1804)	*Byrsonima crassifolia* (1); *Psidium guajava* (1)	Lourido et al. [13]
*Polybia occidentalis* (Olivier,1792)	*Anacardium occidentale* (15); *Cereus jamacaru* (42); *Melocactus bahiensis* (1); *Pilosocereus catingicola* (12); *Plinia cauliflora* (2); *Xiquexique gounellei* (14)	Santos et al. [25]; De Souza et al. [20]; Santos & Presley [24]; Oliveira et al. [23]
*Polybia paulista* H. von Ihering, 1896	*Anacardium occidentale* (10); *Cereus jamacaru* (20); *Couroupita guianensis* (1); *Harrisia adscendens* (30); *Melocactus bahiensis* (1); *Pilosocereus catingicola* (29); *Psidium guajava* (23); *Xiquexique gounellei* (25)	Santos et al. [25]; Santos & Presley [24]; Renne et al. [22]; Field observations (this study)
*Polybia platycephala* Richards, 1978	*Eugenia uniflora* (51); *Mangifera indica* (52); *Psidium guajava* (2)	Souza et al. [21]; Barbosa et al. [12]; Renne et al. [22]
*Polybia punctata* DuBuysson, 1908	*Mangifera indica* (1); *Psidium guajava* (1)	iNaturalist [Table S1, record 29, 30]
*Polybia rejecta* (Fabricius, 1798)	*Mangifera indica* (1); *Morus* sp. (1); *Syzygium cumini* (1)	iNaturalist [Table S1, record 31]; Field observations (this study)
*Polybia scutellaris* (White,1841)	*Mangifera indica* (1); *Syzygium jambos* (32)	Brugger et al. [26]; Barbosa et al. [12]
*Polybia sericea* (Olivier, 1792)	*Anacardium occidentale* (63); *Cereus jamacaru* (46); *Eugenia uniflora* (5); *Harrisia adscendens* (3); *Mangifera indica* (2); *Melocactus bahiensis* (1); *Musa* sp. (4); *Opuntia palmadora* (1); *Pilosocereus catingicola* (194); *Plinia cauliflora* (2); *Psidium guajava* (1); *Syzygium jambos* (1); *Xiquexique gounellei* (11)	Santos et al. [25]; De Souza et al. [20]; Santos & Presley [24]; Souza et al. [21]; Renne et al. [22]; Oliveira et al. [23]; iNaturalist [Table S1, record 32–36]; Field observations (this study)
*Polybia* sp.	*Malus domestica* (3); *Mangifera indica* (9); *Musa* sp. (1); *Plinia cauliflora* (1); *Syzygium cumini* (1)	Barbosa et al. [12]; iNaturalist [Table S1, record 37–42]; Field observations (this study)
*Polybia striata* (Fabricius, 1787)	*Mangifera indica* (1); *Musa* sp. (1); *Plinia cauliflora* (1); *Syzygium cumini* (1)	Oliveira et al. [23]; iNaturalist [Table S1, record 43–45]
*Protonectarina sylveirae* (de Saussure, 1854)	*Anacardium occidentale* (34)	Santos & Presley [24]
*Protopolybia exigua* (de Saussure, 1854)	*Eugenia uniflora* (2)	Souza et al. [21]
*Synoeca chalibea* de Saussure 1852	*Psidium guajava* (1)	iNaturalist [Table S1, record 46]
*Synoeca cyanea* (Fabricius, 1775)	*Anonnaceae* sp. (1); *Citrus* sp (1); *Eugenia uniflora* (1), *Litchi chinensis* (1); *Mangifera indica* (3); *Musa* sp. (3); *Plinia cauliflora* (6); *Psidium guajava* (149); *Rubus idaeus* (1); *Rubus rosifolius* (2); *Spondias purpurea* (1); *Syagrus romanzoffiana* (1); *Vitis* sp. (1)	De Souza et al. [20]; Brugger et al. [11]; Prezoto & Braga [27]; Barbosa et al. [12]; Renne et al. [22]; Oliveira et al. [23]; iNaturalist [Table S1, record 47–67]
*Synoeca ilheensis* Lopes & Menezes, 2017	*Eugenia uniflora* (1); *Mangifera indica* (1)	iNaturalist [Table S1, record 68, 69]
*Synoeca* sp.	*Anacardium occidentale* (2); *Mangifera indica* (1), *Morus* sp. (1); *Psidium guajava* (2)	iNaturalist [Table S1, record 70–74]; Field observations (this study)
*Synoeca surinama* (Linnaeus, 1767)	*Anacardium occidentale* (2); *Carica papaya* (1); *Eriobotrya japonica* (1); *Melastomataceae* sp. (1); *Morus* sp. (1); *Myrcia* sp. (1); *Psidium guajava* (4)	Lourido et al. [13]; iNaturalist [Table S1, record 75–84]; Field observations (this study)
*Synoeca virginea* (Fabricius, 1804)	*Averrhoa carambola* (1); *Mangifera indica* (1); *Myrcia* sp. (1); *Psidium guajava* (2)	Lourido et al. [13]; iNaturalist [Table S1, record 85–87]

## Data Availability

The original contributions presented in this study are included in the article/Supplementary Materials. Further inquiries can be directed to the corresponding author.

## Supplementary Materials

The following supporting information can be downloaded at: https://www.mdpi.com/article/10.3390/insects17040409/s1, Table S1: Record IDs, wasp species, plant species, and URLs of the iNaturalist records included in this study.
