# Supplementary material for: Social Wasps and Fruit Exploitation in Brazil: A Synthesis of Species Records, Resource Use, and Management Implications
Source: Insects. 2026 Apr 10;17(4):409. doi: 10.3390/insects17040409 (PMC13115773; doi:10.3390/insects17040409)
Supplement: Supplementary file 1 [file insects-17-00409-s001.zip › insects-4222719-supplementary.pdf]

Supplementary Table S1: Record IDs, wasp species, plant species, and URLs of the iNaturalist records included in this study.

Record ID	Wasp species	Plant species	iNaturalist URL
1	*Agelaia angulata* (Fabricius, 1804)	*Musa* sp.	https://www.inaturalist.org/observations/234755597
2	*Agelaia angulata* (Fabricius, 1804)	*Musa* sp.	https://www.inaturalist.org/observations/217588800
3	*Agelaia angulata* (Fabricius, 1804)	*Citrus* sp.	https://www.inaturalist.org/observations/208592910
4	*Agelaia multipicta* (Haliday, 1836)	*Malus domestica*	https://www.inaturalist.org/observations/139669745
5	*Agelaia pallipes* (Olivier, 1792)	*Anacardium occidentale*	https://www.inaturalist.org/observations/240698132
6	*Agelaia pallipes* (Olivier, 1792)	*Eriobotrya japonica*	https://www.inaturalist.org/observations/177079794
7	*Agelaia pallipes* (Olivier, 1792)	*Musa* sp.	https://www.inaturalist.org/observations/201592736
8	*Apoica flavissima* Van der Vecht, 1972	*Mangifera indica*	https://www.inaturalist.org/observations/194311498
9	*Apoica* sp.	*Musa* sp.	https://www.inaturalist.org/observations/235640941
10	*Polistes canadensis* (Linnaeus, 1758)	*Eugenia uniflora*	https://www.inaturalist.org/observations/246562843
11	*Polistes canadensis* (Linnaeus, 1758)	*Psidium guajava*	https://www.inaturalist.org/observations/45736494
12	*Polistes canadensis* (Linnaeus, 1758)	*Xiquexique gounellei*	https://www.inaturalist.org/observations/149160626
13	*Polistes canadensis* (Linnaeus, 1758)	*Spondias purpurea*	https://www.inaturalist.org/observations/147109293
14	*Polistes carnifex* (Fabricius, 1775)	*Psidium guajava*	https://www.inaturalist.org/observations/201420975
15	*Polistes lanio* (Fabricius, 1775)	*Pilosocereus arrabidae*	https://www.inaturalist.org/observations/290292557
16	*Polistes* sp.	*Musa* sp.	https://www.inaturalist.org/observations/246699385
17	*Polistes versicolor* (Olivier, 1792)	*Momordica charantia*	https://www.inaturalist.org/observations/199305534
18	*Polybia chrysothorax* (Lichtenstein, 1796)	*Mangifera indica*	https://www.inaturalist.org/observations/204921489
19	*Polybia ignobilis* (Haliday, 1836)	*Musa* sp.	https://www.inaturalist.org/observations/228478946
20	*Polybia ignobilis* (Haliday, 1836)	*Mangifera indica*	https://www.inaturalist.org/observations/226398210
21	*Polybia ignobilis* (Haliday, 1836)	*Musa* sp.	https://www.inaturalist.org/observations/220707900
22	*Polybia ignobilis* (Haliday, 1836)	*Spondias tuberosa*	https://www.inaturalist.org/observations/219118261
23	*Polybia ignobilis* (Haliday, 1836)	*Plinia cauliflora*	https://www.inaturalist.org/observations/20812064
24	*Polybia ignobilis* (Haliday, 1836)	*Plinia cauliflora*	https://www.inaturalist.org/observations/195791446
25	*Polybia jurinei* de Saussure, 1854	*Theobroma cacao*	https://www.inaturalist.org/observations/223844982
26	*Polybia jurinei* de Saussure, 1854	*Musa* sp.	https://www.inaturalist.org/observations/214636997
27	*Polybia jurinei* de Saussure, 1854	*Citrullus lanatus*	https://www.inaturalist.org/observations/212791736
28	*Polybia jurinei* de Saussure, 1854	*Mangifera indica*	https://www.inaturalist.org/observations/193315310
29	*Polybia punctata* DuBuysson, 1908	*Mangifera indica*	https://www.inaturalist.org/observations/260850221
30	*Polybia punctata* DuBuysson, 1908	*Psidium guajava*	https://www.inaturalist.org/observations/199138171
31	*Polybia rejecta* (Fabricius, 1798)	*Mangifera indica*	https://www.inaturalist.org/observations/199228219
32	*Polybia sericea (Olivier, 1792)*	*Plinia cauliflora*	https://www.inaturalist.org/observations/254201873
33	*Polybia sericea (Olivier, 1792)*	*Musa* sp.	https://www.inaturalist.org/observations/228478512
34	*Polybia sericea (Olivier, 1792)*	*Syzygium jambos*	https://www.inaturalist.org/observations/216561121
35	*Polybia sericea (Olivier, 1792)*	*Musa* sp.	https://www.inaturalist.org/observations/214636992
36	*Polybia sericea (Olivier, 1792)*	*Musa* sp.	https://www.inaturalist.org/observations/213223802
37	*Polybia* sp.	*Musa* sp.	https://www.inaturalist.org/observations/213820341
38	*Polybia* sp.	*Malus domestica*	https://www.inaturalist.org/observations/213523589
39	*Polybia* sp.	*Malus domestica*	https://www.inaturalist.org/observations/213315302
40	*Polybia* sp.	*Malus domestica*	https://www.inaturalist.org/observations/212795114

41	*Polybia* sp.	*Plinia cauliflora*	https://www.inaturalist.org/observations/210904270
42	*Polybia* sp.	*Syzygium cumini*	https://www.inaturalist.org/observations/203942340
43	*Polybia striata* (Fabricius, 1787)	*Musa* sp.	https://www.inaturalist.org/observations/222740835
44	*Polybia striata* (Fabricius, 1787)	*Mangifera indica*	https://www.inaturalist.org/observations/205630898
45	*Polybia striata* (Fabricius, 1787)	*Syzygium cumini*	https://www.inaturalist.org/observations/199453684
46	*Synoeca chalibea* de Saussure 1852	*Psidium guajava*	https://www.inaturalist.org/observations/99392131
47	*Synoeca cyanea* (Fabricius, 1775)	*Psidium guajava*	https://www.inaturalist.org/observations/247782282
48	*Synoeca cyanea* (Fabricius, 1775)	*Citrus* sp.	https://www.inaturalist.org/observations/248110218
49	*Synoeca cyanea* (Fabricius, 1775)	*Psidium guajava*	https://www.inaturalist.org/observations/246245722
50	*Synoeca cyanea* (Fabricius, 1775)	*Vitis* sp.	https://www.inaturalist.org/observations/224890783
51	*Synoeca cyanea* (Fabricius, 1775)	*Rubus rosifolius*	https://www.inaturalist.org/observations/224169052
52	*Synoeca cyanea* (Fabricius, 1775)	*Plinia cauliflora*	https://www.inaturalist.org/observations/292213584
53	*Synoeca cyanea* (Fabricius, 1775)	*Eugenia uniflora*	https://www.inaturalist.org/observations/318283416
54	*Synoeca cyanea* (Fabricius, 1775)	*Plinia cauliflora*	https://www.inaturalist.org/observations/185092378
55	*Synoeca cyanea* (Fabricius, 1775)	*Psidium guajava*	https://www.inaturalist.org/observations/151377982
56	*Synoeca cyanea* (Fabricius, 1775)	*Rubus rosifolius*	https://www.inaturalist.org/observations/151770026
57	*Synoeca cyanea* (Fabricius, 1775)	*Litchi chinensis*	https://www.inaturalist.org/observations/255431680
58	*Synoeca cyanea* (Fabricius, 1775)	*Plinia cauliflora*	https://www.inaturalist.org/observations/105504294
59	*Synoeca cyanea* (Fabricius, 1775)	*Syagrus romanzoffiana*	https://www.inaturalist.org/observations/216401029
60	*Synoeca cyanea* (Fabricius, 1775)	*Musa* sp.	https://www.inaturalist.org/observations/214767208
61	*Synoeca cyanea* (Fabricius, 1775)	*Musa* sp.	https://www.inaturalist.org/observations/214636993
62	*Synoeca cyanea* (Fabricius, 1775)	*Musa* sp.	https://www.inaturalist.org/observations/213758617
63	*Synoeca cyanea* (Fabricius, 1775)	*Psidium guajava*	https://www.inaturalist.org/observations/213431990
64	*Synoeca cyanea* (Fabricius, 1775)	*Rubus idaeus*	https://www.inaturalist.org/observations/211814432
65	*Synoeca cyanea* (Fabricius, 1775)	*Psidium guajava*	https://www.inaturalist.org/observations/205519578
66	*Synoeca cyanea* (Fabricius, 1775)	*Psidium guajava*	https://www.inaturalist.org/observations/194446865
67	*Synoeca cyanea* (Fabricius, 1775)	Anonnaceae	https://www.inaturalist.org/observations/194215105
68	*Synoeca ilheensis* Lopes & Menezes, 2017	*Mangifera indica*	https://www.inaturalist.org/observations/221524347
69	*Synoeca ilheensis* Lopes & Menezes, 2017	*Eugenia uniflora*	https://www.inaturalist.org/observations/48565901
70	*Synoeca* sp.	*Psidium guajava*	https://www.inaturalist.org/observations/221357813
71	*Synoeca* sp.	*Psidium guajava*	https://www.inaturalist.org/observations/207690557
72	*Synoeca* sp.	*Morus* sp.	https://www.inaturalist.org/observations/200545080
73	*Synoeca* sp.	*Anacardium occidentale*	https://www.inaturalist.org/observations/197158678
74	*Synoeca* sp.	*Anacardium occidentale*	https://www.inaturalist.org/observations/193596258
75	*Synoeca surinama* (Linnaeus, 1767)	*Anacardium occidentale*	https://www.inaturalist.org/observations/245136372
76	*Synoeca surinama* (Linnaeus, 1767)	*Anacardium occidentale*	https://www.inaturalist.org/observations/243722867
77	*Synoeca surinama* (Linnaeus, 1767)	*Psidium guajava*	https://www.inaturalist.org/observations/99392131
78	*Synoeca surinama* (Linnaeus, 1767)	Melastomataceae	https://www.inaturalist.org/observations/257148131
79	*Synoeca surinama* (Linnaeus, 1767)	*Myrcia* sp.	https://www.inaturalist.org/observations/152131999
80	*Synoeca surinama* (Linnaeus, 1767)	*Psidium guajava*	https://www.inaturalist.org/observations/204140397
81	*Synoeca surinama* (Linnaeus, 1767)	*Carica papaya*	https://www.inaturalist.org/observations/201463069
82	*Synoeca surinama* (Linnaeus, 1767)	*Psidium guajava*	https://www.inaturalist.org/observations/200911074
83	*Synoeca surinama* (Linnaeus, 1767)	*Eriobotrya japonica*	https://www.inaturalist.org/observations/199122381
84	*Synoeca surinama* (Linnaeus, 1767)	*Psidium guajava*	https://www.inaturalist.org/observations/192964457

85	*Synoeca virginea (Fabricius, 1804)*	*Psidium guajava*	https://www.inaturalist.org/observations/243861181
86	*Synoeca virginea (Fabricius, 1804)*	*Myrcia* sp.	https://www.inaturalist.org/observations/103218447
87	*Synoeca virginea (Fabricius, 1804)*	*Mangifera indica*	https://www.inaturalist.org/observations/193315453
